# Early vs late fusion of qMIDS with machine learning based classifiers for oral cancer diagnosis from gene expression data

**DOI:** 10.1371/journal.pcbi.1014888

**Published:** 2026-10-28

**Authors:** Ferid Ben Ali, Iosif Mporas, Vaishnavi Balaji, Dickson Ibaranyanakaye, Barbara Carey, Muy-Teck Teh, Sola Adeleke

**Affiliations:** 1 School of Physics, Engineerng and Computer Science, University of Hertfordshire, Hatfield, United Kingdom; 2 Curenetics Ltd, London, United Kingdom; 3 Guy’s and St Thomas’ NHS Foundation Trust, London, United Kingdom; 4 Centre for Oral Immunobiology and Regenerative Medicine, Institute of Dentistry, Barts and The London School of Medicine and Dentistry, Queen Mary University of London, London, United Kingdom; Burnet Institute, AUSTRALIA

## Abstract

Different fusion architectures of the quantitative Malignancy Index Diagnostic System (qMIDS) method with machine learning classifiers for oral cancer diagnosis from gene expression data are investigated. The proposed fusion architectures are tested on geographically and ethnically diverse datasets and the evaluation results showed performance improvements with late fusion, which significantly outperformed early fusion in more matched model configurations than the reverse in every cohort, although at the best-performing configuration the two architectures were statistically indistinguishable in most cohorts. Inter-dataset analysis showed robustness of the proposed architectures when the training/test samples are from different populations.

## Author summary

The study tests two fusion designs of the qMIDS gene-expression method combined with machine-learning classifiers for oral cancer diagnosis. Evaluations across geographically and ethnically diverse cohorts show that late fusion performs best overall, although a direct statistical comparison of the two designs shows that its advantage over early fusion, while consistent in direction, is modest and does not reach statistical significance at the best-performing setting in most cohorts. Cross-dataset experiments (training and testing on different populations) indicate the architectures are robust to population shifts.

## Introduction

Oral cancer, of which oral squamous cell carcinoma (OSCC) accounts for approximately 90% of cases, is a major global health concern affecting the lips, tongue, gums and cheek lining [1,2]. Primary risk factors include tobacco use, excessive alcohol consumption and HPV infection [1,3], and incidence is age- and sex-dependent, rising after 50 years and 2–3 times more common in men [1,4]. Incidence rates vary considerably by region, from ~1.5 per 100,000 in China to ~9.9 in India and ~4.5-4.6 in the UK and USA [5,6]. Despite treatment advances, the overall 5-year survival rate remains ~50%, ranging from 90% for early-stage to 30% for late-stage disease [1,2,7], underscoring the importance of early detection.

Early detection of high-risk oral premalignant disorder (OPMD) lesions and/or early-stage OSCC is crucial in improving outcomes and reducing mortality [1,2]. Current diagnostic methods based on histopathology cannot reliably predict which OPMDs lesions will progress to oral cancer [1]. Recent research efforts have focused on developing novel molecular diagnostic tools for the early detection of high-risk oral lesions. For example, a recent study investigated serum metabolomics in a large patient cohort consisting of 241 healthy volunteers and 578 OSCC patients with machine learning (ML) identified distinct metabolic patterns correlated with OSCC [8]. Another study analysed 6 microarray databases and identified a 4-gene signature predictive of oral cancer recurrence [9]. Unfortunately, most of these studies involved only a single validation cohort, thereby their assay generalisability remains unknown.

We have previously conducted one of the largest international multicohort validation studies [10] using the qMIDS method [11] that involves measuring mRNA of 16 genes from a 1 mm minimally invasive tissue biopsy for the risk stratification of OPMD and OSCC in over 500 samples from geographical and ethnically diverse patients from the UK, India, and China. The qMIDS assay involves using reverse transcription quantitative polymerase chain reaction (RT-qPCR) to measure a panel of 16 genes associated with a key driver oncogene FOXM1, implicated in the regulation of cell proliferation, differentiation, aging, genomic instability, epigenetics, stem cell reprogramming, stromal and immune modulation. FOXM1 transcription factor has been shown to be amongst the top upregulated oncogenes across 39 cancer types and is a major predictor of poor cancer prognosis [12]. We have demonstrated in our previous studies that qMIDS robustly quantifies a universal FOXM1-driven oncogenic program in OSCC which transcends ethnicity, age, gender and geographic origins [10,11,13].

The qMIDS test requires only a tiny tissue sample (~1 mm), in stark contrast to conventional surgical biopsies that typically extract >5–10 mm of tissue and may necessitate general anaesthesia for patients unable to tolerate local injections. This minimally invasive approach not only reduces patient discomfort but also significantly shortens the time from biopsy to result—delivering molecular insights within 90 minutes, compared to over a week for histopathology. The small sample size enables rapid punch biopsies and allows for multiple-site sampling in patients with widespread oral field changes, reducing the risk of misdiagnosis. While still invasive, tissue biopsy offers a more site-specific and reliable specimen format than liquid biopsies (e.g., saliva or blood), which often suffer from poor sensitivity and cannot localise lesions. Ultimately, tissue-based diagnostics remain essential for confirming malignancy and guiding surgical intervention [14].

The current qMIDS method relies on a static algorithm trained on a limited dataset, which may reduce its diagnostic performance in populations beyond those we have previously studied. To address this limitation, integrating machine learning (ML) is essential to improve adaptability, generalisability, and diagnostic efficiency across diverse demographic groups. This article therefore investigates the fusion of the qMIDS [11] method with ML classifiers for oral cancer diagnosis, leveraging gene expression data we have already curated from multiple cohorts to develop a more accurate, robust, and population-inclusive diagnostic model.

## Related works

In [15] classification between oral cancer patients and healthy controls using two microarray datasets GSE30784 and GSE37991 from NCBI Gene Expression Omnibus (GEO) repository [16] was presented. The datasets were merged resulting in 292 patient samples with 12,192 common gene features. The most important genes were selected using the rank product method [17] which detected differentially expressed genes, with ranking score cut-off equal to 0.05 to select the most important gene expressions resulting in 3,542 up and down regulated genes. The Significance Analysis of Microarray (SAM) technique [18] was then applied to remove the up and down regulated genes with low changes in their expressions of, resulting in 26 gene features. Classification was then performed using five classifiers: Naive Bayes (NB), Bayesian Network (BN), Decision Trees [19] with AdaBoost meta-learners, Support Vector Machine (SVM) [20], Artificial Neural Network (ANN) and Random Forests (RF) [19] with 10-fold cross validation. The best performing classifier was RF with 89.3% accuracy.

In [21] oral cancer type identification using convolutional neural networks on RNA-seq gene expressions for feature extraction was proposed. RNA sequencing data from OSCC tissues from various sites, including oral, non-oral head and neck, oesophageal, and cervical regions, were obtained from The Cancer Genome Atlas (TCGA) [22]. Oesophageal and cervical OSCCs were classified with accuracies of 99% and 98%, respectively, and 83% of OSCC samples were correctly diagnosed, while 51% of the non-oral cancer samples were classified correctly.

In [23] 10-fold cross validation and hold cross validation from RNA-seq dataset were compared in oral cancer stage classification using k-nearest neighbors (kNN) [24], SVM and RF. The data consisted of 1,505 oral cancer samples obtained from the International Cancer Genome Consortium (ICGC) [25]. Mean imputation and dropping missing values were performed to handle missing values in the data followed by data normalization. The best performing classifier was kNN with 84% accuracy and 84% F1 score.

In [26] the authors identified potential biomarkers for early detection of invasive OSCC by comparing primary OSCC, oral dysplasia, and healthy control from microarray data. Forward and stepwise logistic regression analyses identified 10 successive combinations of genes the expressions of which differentiated OSCC from controls. The models were validated using external microarray datasets resulting in sensitivity and specificity above 95%.

In [27] classification between OSCC and healthy controls was performed using a microarray dataset and SVM. A 25-gene signature specific to OSCC was identified, which effectively distinguished healthy controls from OSCC samples. The SVM model achieved 96% accuracy with 10-fold cross-validation and an average accuracy of 87% across three independent validation sets.

In [28] the authors identified seven genes involved in the progression from oral leukoplakia (OL) to OSCC and evaluated the diagnostic performance using microarray data across twelve machine learning algorithms. The best performing machine learning algorithm was SVM with AUC equal to 0.749.

In [29] the authors proposed a 24-gene signature to classify OSCC stages and predict survival outcomes. Using GSE41613 [30] dataset for training and GSE42743 [30] and GSE26549 [31] datasets for validation, patients were grouped according to the low and high tumor-node-metastasis (TNM) staging system. Disparity index analysis was used to identify signature genes, and classification performance was evaluated using five-fold cross-validation with accuracy equal to 89.7%. The model was validated on GSE42743 with AUC equal to 0.994 and on GSE26549 with AUC equal to 0.984.

In [10], Teh et al. (2022) developed and validated the qMIDS using RT-qPCR on a 14-gene panel applied to over 500 oral tissue samples across three countries. The model achieved high diagnostic performance in three cohorts (AUC = 0.945 for UK cohort, AUC = 0.928 for Indian cohort and AUC = 0.932 for Chinese cohort) in distinguishing OSCC from OPMDs and normal tissues.

In [32], authors classified OSCC cases from controls and OPMDs using Univariate fractional polynomial (FP) models [33]. 180 samples (60 OSCC patients, 60 controls, and 60 OPMD patients) were used in the study with seven transcriptomic markers measured with qPCR. The performance of FP models for OSCC versus OPMD is AUC = 0.80, for OSCC versus controls is AUC = 0.87, and for OPMD versus controls is AUC = 0.78.

Table 1 summarizes the bibliography on oral cancer identification from gene expression data.

**Table 1:** Oral Cancer Diagnosis Methods using Gene Expression Data.

Paper	Dataset	Data type	Classes	Classifier	Performance
[15]	GEO [22]	Microarray	oral cancer patients and healthy controls	NB [34], DT [19], SVM [20], ANN [35], RF [19]	accuracy: 89.3%
[21]	TCGA [22]	RNA-seq	oral, non-oral head and neck, oesophageal, cervical regions	CNN [36]	Oesophageal OSCCs 99% accuracy cervical OSCCs 98% accuracy
[23]	ICGC [25]	RNA-seq	oral cancer stage	kNN [24], SVM, RF	accuracy: 84%
[26]	[26]	Microarray	OSCC vs healthy control	Forward and stepwise LR	sensitivity and specificity above 95%
[27]	[28]	Microarray	OSCC vs healthy control	SVM	accuracy: 87%
[28]	GEO	Microarray	oral leukoplakia vs OSCC	12 ML methods	AUC: 0.749
[29]	GEO	Microarray	OSCC stages	disparity index	accuracy: 89.7%
[10]	[10]	qPCR	OSCC vs OPMD, OSCC vs controls, OPMD vs controls	qMIDS	AUC: 0.80, AUC: 0.87, AUC: 0.78
[32]	Linko Medical Center of Chang Gung Memorial Hospital	qPCR	OSCC vs OPMD, OSCC vs controls, OPMD vs controls	univariate FP	AUC: 0.80, AUC: 0.87, AUC: 0.78

## Materials and methods

### Evaluation data

Brief information about the evaluated datasets for oral cancer diagnosis, obtained from [10], is tabulated in Table 2.

**Table 2:** Evaluated Oral Cancer Datasets.

Cohort Origin	OSCC Dataset	#Cancer samples	#Healthy samples	#Total samples	#Gene Features
UK	UK	135	174	309	14
India	IN-N	23	25	48	14
	IN-S	11	22	33	14
China	CN	11	24	35	14

The Indian dataset consists of two subsets, from North India (IN-N) and South India (IN-S). The samples in the datasets consist of relative quantitative information of 14 target genes (HOXA7, CENPA, NEK2, DNMT1, INHBA, FOXM1, TOP2A, BIRC5, MMP13, CXCL8, NR3C1, IVL, CBX7, and S100A16) measured by RT-qPCR from each sample, including quality features that assess mRNA sample yield (mean cycle threshold of two reference genes, YAP1 and POLR2A, measured in each sample), according to the qMIDS method [11]. These genes were initially selected from clinical tissue samples from the UK and Norway [11] and subsequently validated in samples from the UK, China, and India, demonstrating the generalisability of these genes in geographic and ethically diverse populations [10]. The two reference genes, YAP1 and POLR2A, were selected on the basis of a prior systematic screen of candidate endogenous control genes across a panel of normal oral keratinocytes (primary cells and cell lines), normal gingival and buccal mucosa, normal tongue, oral dysplasias, and HNSCC tissues, in which YAP1 and POLR2A were found to be consistently and stably expressed [37]. In accordance with our previously published methodology [11], these two reference genes were used both to normalise the 14 target-gene expression values and as a correction factor within the qMIDS malignancy index calculation itself (Eq 1).

### RT-qPCR data preprocessing

All RT-qPCR data were processed using a standardised pipeline to ensure consistency across cohorts and reproducibility of the downstream machine-learning analyses. Raw cycle threshold (Ct) values were first inspected for quality and technical outliers. Gene-level expression was then normalised using the ΔΔCt method referenced to the two endogenous control genes described above (YAP1 and POLR2A) [37], and the resulting normalised expression values were transformed onto a log_2_ scale to stabilise variance across the dynamic range of expression and reduce the influence of extreme values. The processed molecular features – the 14 diagnostic genes and the qMIDS malignancy index (MI) score – were then assembled into a unified feature matrix used as input to the machine-learning workflow described below.

No explicit cross-cohort batch correction (e.g., ComBat) was applied to the UK, IN-N, IN-S and CN datasets. This was a deliberate choice: removing cohort-level shifts computationally risks eliminating genuine biological differences associated with geography, ethnicity, environmental exposure and population-specific tumour biology, which are themselves of clinical interest when evaluating the cross-population transferability of qMIDS (see Inter-Dataset Analysis and Discussion). Preserving these variations allows the models evaluated here to be tested on the true, unadjusted between-population signal rather than on an artefactually homogenised one.

Technical variability in RT-qPCR measurements, arising for example from differences in RNA yield, pipetting precision, amplification efficiency and reagent lots, can introduce noise into ΔCt/ΔΔCt values and, if unmitigated, can shift the apparent expression distribution of the diagnostic genes and affect classifier decision boundaries. Several elements of the present workflow reduce this risk: ΔΔCt normalisation against two stable reference genes dampens run-to-run fluctuations; the log_2_ transformation further stabilises variance across the expression range; and the use of nested cross-validation across multiple classifiers reduces sensitivity to technical perturbations in any single run. The improved robustness observed for the late-fusion architecture (see Results) is consistent with this: by aggregating predictions across two complementary feature representations (the gene-expression-based classifier output and the qMIDS MI score) rather than relying on either alone, late fusion is inherently less sensitive to fluctuations in any one qPCR-derived measurement.

### qMIDS method

The qMIDS method computes and translates gene expression signatures via an algorithm (1) into a malignancy index (MI) scoring system [11]. As shown in Fig 1, a 1–2 mm tissue sample is acquired from the oral cavity and is digested into a lysate.

**Fig 1:**
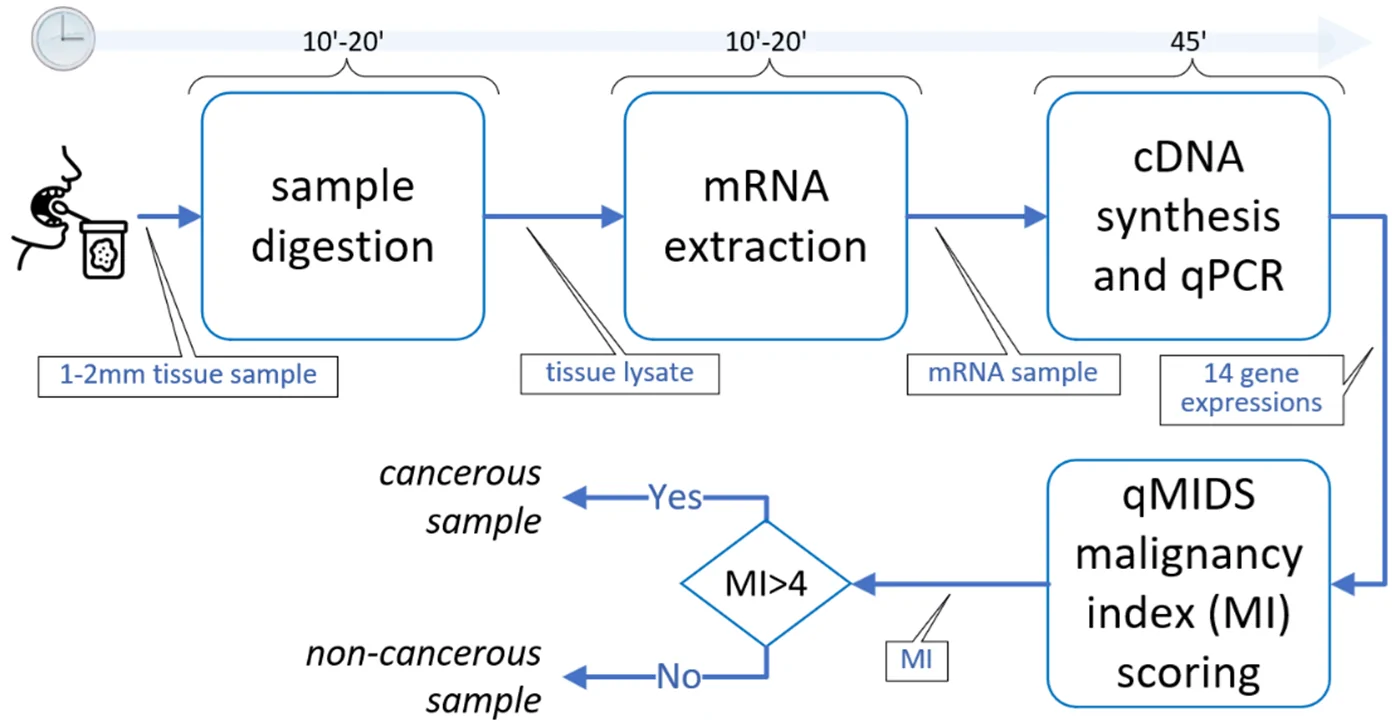
qMIDS method pipeline for oral cancer identification.

Then, mRNA is purified using Dynabeads [38] from the tissue lysate, and the extracted mRNA sample is used directly in a one-step RT-qPCR reaction to generate 14 gene expression data, plus 2 reference genes. The qMIDS method converts the 14 target gene mRNA levels into a single qMIDS MI for clinically meaningful quantitative scores in cancer diagnosis and tumour stratification, as follows:


$$\begin{array}{c} MI=\sum_{i=1}^{g}{\left|\log_{2}\left(\frac{T_{g}\cdot T_{g}d}{T_{g}\cdot T_{d}}\right)\right|}_{i}\cdot \log_{2}\left(Q_{4}\cdot (Q_{3}-Q_{1})\right) \end{array}$$ (1)

where T represents the target gene mRNA transcripts (normalised against 2 reference genes), $T_{g}$ represents the sum of g number of target gene mRNA transcripts measured in each cDNA sample, $T_{d}$ represents a median value of T derived from a control set of 8 independent healthy primary normal human oral keratinocytes, $T_{g}d$ represents the sum of the g number of $T_{d}$ values. *Q*_1_, *Q*_3_ and *Q*_4_ represent the first (25%), third (75%) and fourth (100%) rank quartile of the x number of target gene absolute fractional log2 ratio distribution values within each cDNA sample. MI values are proportionately calculated to fit a linear scale. To decide whether an oral tissue sample is cancerous or not, a threshold at MI = 4 has empirically been selected. The entire workflow takes less than 90 minutes from tissue to results. Further details can be found in [10,11].

In more intuitive terms, the qMIDS algorithm calculates the MI by comparing the gene-expression pattern of a test sample with that typically seen in healthy oral tissue. For each of the 14 target genes, the sample’s normalised expression is compared with a reference median value derived from healthy tissue via a log_2_ ratio; these 14 individual gene-level differences are then summed to give an overall measure of how far the sample’s expression profile deviates from the healthy reference. This sum is then scaled by a term that captures how unevenly this deviation is distributed across the 14 genes, using the first quartile (*Q*_1_), third quartile (*Q*_3_) and maximum (*Q*_4_) of the per-gene deviation values – a sample in which a small subset of genes is very strongly dysregulated is treated differently from one in which all genes are moderately and uniformly shifted. The resulting product is linearly rescaled to give the final MI. Intuitively, a higher MI indicates that a sample’s gene-expression profile both deviates more strongly from, and is more unevenly distributed than, that of healthy tissue – a pattern that more closely resembles malignant behaviour.

### Fusion methodology

#### Early fusion.

The block diagram of the early fusion architecture for oral cancer diagnosis is illustrated in Fig 2. Let us consider a genomic dataset $X\in {\mathbb{R}}^{N\times M}$, where N is the number of samples of the dataset and M is the number of gene expressions per sample. The dataset is initially preprocessed, with preprocessing consisting of isolating the 14 targeted gene expressions.

**Fig 2:**
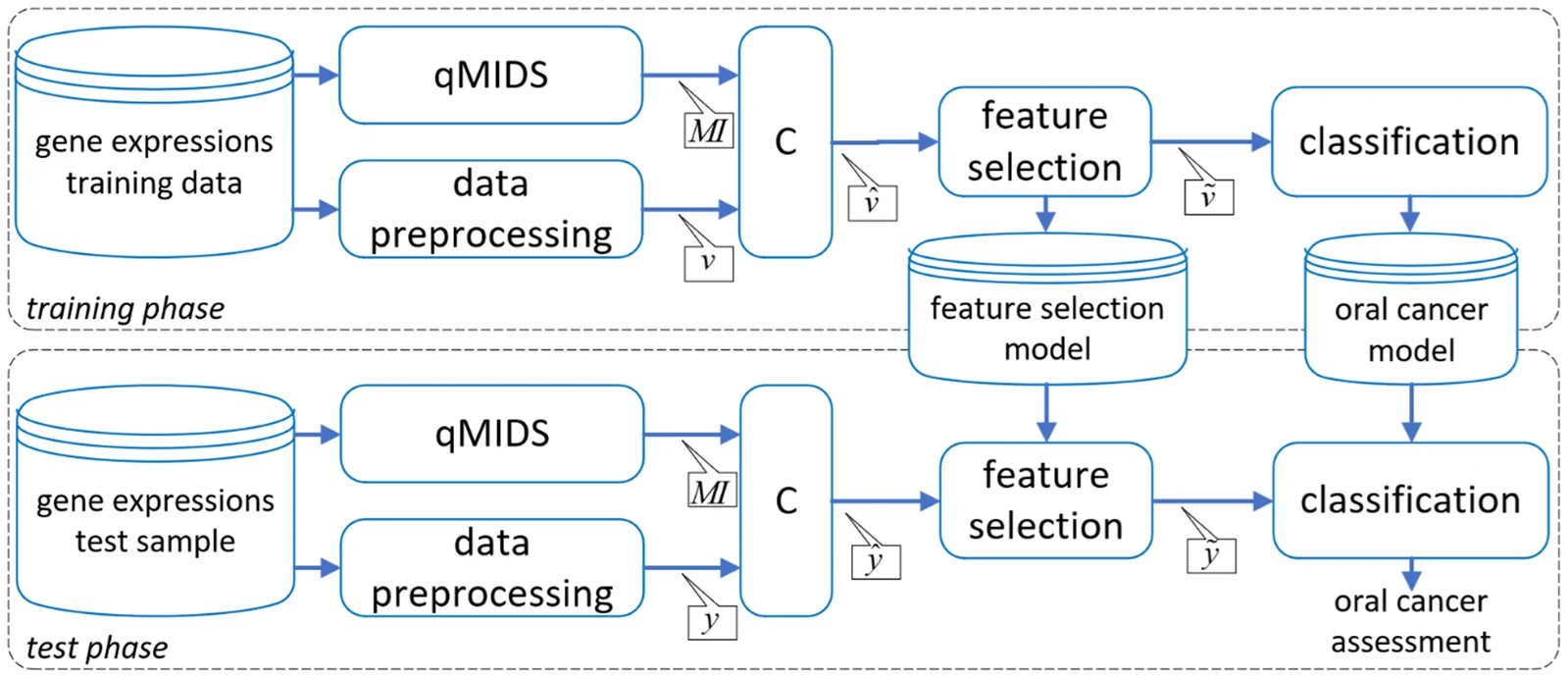
Early Fusion Block Diagram.

In parallel, the outcome MI of the qMIDS method is estimated, where $MI\in {\mathbb{R}}^{N\times 1}$, and is concatenating (block ‘C’) with the 14 target genes v where $v\in {\mathbb{R}}^{N\times 14}$, resulting in v where $\hat{v}\in {\mathbb{R}}^{N\times 15}$. The MI score is treated identically to the 14 individual gene-expression features from this point onward: it is not weighted, scaled or preprocessed differently, and it is equally eligible to be selected or discarded by the feature selection algorithm below. A feature selection algorithm is subsequently applied resulting in $\tilde{v}\in {\mathbb{R}}^{N\times K}$, where 1⩽K⩽15 is the K-th dimensional representation of the 15 features. After that a classification algorithm is used to train an oral cancer identification model.

During the test phase, a new unknown oral tissue sample is preprocessed and early fusion using the same process as in the training phase is followed resulting in the early fusion feature vector $\hat{y}\in {\mathbb{R}}^{1\times 15}$. The trained feature selection model is then used to extract a new vector representation $\tilde{y}\in {\mathbb{R}}^{1\times K}$ with K being the reduced dimensionality representation of the test sample. Finally, the oral cancer classification model is used to label the unknown test sample as oral cancer or non-cancerous.

#### Late fusion.

The block diagram of the late fusion architecture for oral cancer diagnosis is illustrated in Fig 3. The training gene expressions data, $X\in {\mathbb{R}}^{N\times M}$, are initially preprocessed selecting only the 14 target genes v, where $v\in {\mathbb{R}}^{N\times 14}$, followed by feature selection resulting in $\tilde{v}\in {\mathbb{R}}^{N\times K}$, where 1⩽K⩽14 is the K-th dimensional representation of the 14 gene features. After that a classification algorithm is used to train an oral cancer identification model and the class probabilities, $\hat{v}\in {\mathbb{R}}^{N\times 2}$, are concatenated (block ‘C’) with qMIDS’ MI score, where $MI\in {\mathbb{R}}^{N\times 1}$. The new feature vector $\overset{ˇ}{v}\in {\mathbb{R}}^{N\times 3}$ is then used to train a late fusion meta-classifier.To avoid information leakage, the base classifier’s class probabilities used to train the meta-classifier were obtained via proper stacking, i.e., out-of-fold predictions generated when each training sample was held out of the base classifier’s own training fold, rather than predictions from a base classifier trained and evaluated on the same fold. Probability calibration of the base and meta-classifiers was additionally assessed via calibration curves for the best-performing model in each cohort and each train/test combination (Supporting Information, S2 Fig).

**Fig 3:**
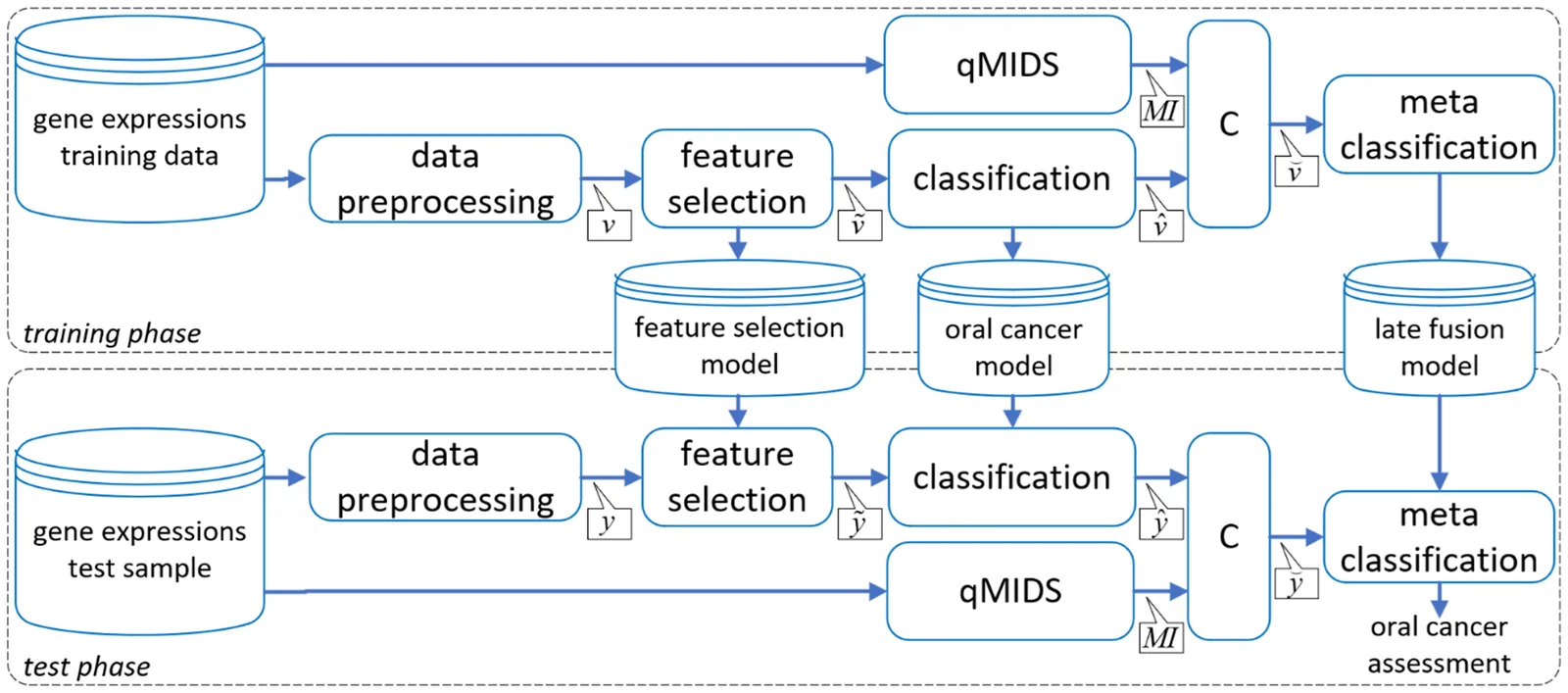
Late Fusion Block Diagram.

During the test phase, a test oral tissue sample follows the same preprocessing, feature extraction and classification steps as in the training phase and using the corresponding trained models, resulting in the vectors $y\in {\mathbb{R}}^{1\times 14}$, $\tilde{y}\in {\mathbb{R}}^{1\times K}$ and $\hat{y}\in {\mathbb{R}}^{1\times 2}$, respectively. The oral cancer recognition probabilities $\hat{y}$ are concatenated with qMIDS’ score $MI\in {\mathbb{R}}^{N\times 1}$ and the late fusion feature vector, $\overset{ˇ}{y}\in {\mathbb{R}}^{1\times 3}$, is processed by the oral cancer meta-classification model to label the unknown test sample as oral cancer or non-cancerous.

#### Feature selection.

Two different algorithms for dimensionality reduction were evaluated, namely the Minimum Redundancy Maximum Relevance (mRMR) [39] and the Recursive Feature Elimination with SVM (SVM-RFE) [40]. Oral cancer identification was evaluated on the top K={2,3,5,7,10,12,14,15} features for early fusion and on the top K={2,3,5,7,10,12,14} features for late fusion approach. mRMR and SVM-RFE were chosen because they represent two complementary feature-selection paradigms: mRMR is a filter method that ranks genes by relevance to the diagnostic label while explicitly penalising redundancy between co-selected genes, independently of any classifier, whereas SVM-RFE is a wrapper method that iteratively removes the least discriminative feature(s) with respect to a specific classifier’s decision boundary. Given the small number of candidate features (14–15) and the moderate sample sizes of some cohorts, evaluating both a classifier-agnostic and a classifier-specific selection strategy allowed us to assess whether the identified reduced gene panels were sensitive to the choice of selection paradigm, rather than assuming a single method would be appropriate across all four cohorts.

#### Classification.

Eight ML algorithms for classification were used, namely the Support Vector Machine (SVM) [20] with linear, Radial Basic Function (RBF) and polynomial kernels, Random Forest (RF) [41], k-Nearest Neighbors (kNN) [42], Logistic Regression (LR) [43], AdaBoost (AB) [44], Gradient Boosting (GB) [45], Extra Trees (ET) [19], Linear Discriminant Analysis (LDA) [46] and XGBoost (XGB) [47]. This deliberately broad panel of classifiers was evaluated, rather than a single a-priori chosen algorithm, because no single classifier is guaranteed to perform best across cohorts that differ in sample size, class balance and feature dimensionality; several of the algorithms are related (e.g., AdaBoost, Gradient Boosting and XGBoost are all boosted ensembles of weak learners, and Random Forest and Extra Trees are both bagged tree ensembles) but differ in bias-variance trade-offs that can matter for the small cohorts (IN-S, CN) evaluated here. Reporting the best-performing model per cohort, rather than fixing a single classifier a priori, therefore reflects standard practice for benchmarking on heterogeneous, small-to-moderate sized clinical datasets.

Hyperparameters for every classifier were tuned using grid search with stratified five-fold cross-validation (GridSearchCV), nested within the outer ten-fold cross-validation used for performance evaluation (i.e., hyperparameter tuning used only the training folds of each outer split, and was re-run independently within each outer fold, so that no test-fold information was used for model selection). Class weights were balanced for classifiers that support this option, to account for the moderate class imbalance present in some cohorts (Table 2). The base configuration and hyperparameter search space evaluated for each classifier are summarised in Table 3.

**Table 3:** Base configuration and hyperparameter grid search space for each classifier.

Classifier	Base configuration	Hyperparameter grid (search space)
SVM (linear)	SVC(kernel= ’linear’, class_weight=’balanced’)	C: [0.001, 0.01, 0.1, 0.5, 1, 2, 5]
SVM (RBF)	SVC(kernel=’rbf’, class_weight=’balanced’)	C: [0.1, 1, 10]; gamma: [0.001, 0.01, 0.1, 0.5, 1, 2, 5]
Random Forest	RandomForestClassifier(class_weight=’balanced’)	n_estimators: [50, 100, 200]
Logistic Regression	LogisticRegression(max_iter=10000, class_weight=’balanced’)	C: [0.1, 1, 10]
kNN	KNeighborsClassifier()	n_neighbors: [3, 5, 7]
Gradient Boosting	GradientBoostingClassifier()	n_estimators: [50, 100, 200]; learning_rate: [0.01, 0.1, 0.2]
AdaBoost	AdaBoostClassifier()	n_estimators: [100, 200, 500]
Extra Trees	ExtraTreesClassifier(class_weight=’balanced’)	n_estimators: [50, 100, 200]; max_depth: [None, 10, 20]
LDA	LinearDiscriminantAnalysis()	(default parameters; no tuning grid)
XGBoost	XGBClassifier(eval_metric= ’logloss’)	n_estimators: [50, 100, 200, 500]; learning_rate: [0.01, 0.1, 0.2]

Performance evaluation used ten-fold cross-validation, with performance metrics averaged across the ten outer folds; feature selection (mRMR/SVM-RFE) was nested within each outer fold, i.e., re-fitted on the training portion of each fold only. As described above, the five-fold inner cross-validation used for hyperparameter tuning was itself nested within each outer training fold, so that the outer test fold was never used for either feature selection or hyperparameter optimisation.

## Results

The two fusion methodologies were compared to qMIDS and to ML based classification, using the four datasets presented in the Materials and methods section, following the ten-fold cross-validation protocol and nested hyperparameter tuning procedure described in the Materials and methods section (Classification).

### Early fusion

The early fusion results for each of the four datasets are tabulated in Table 4. The qMIDS based and the best ML based results, i.e., without fusion, are highlighted in grey. The best results without feature selection (FS) and with the best FS are tabulated as well.

**Table 4:** Oral cancer identification results with early fusion (results without fusion shown in grey).

Cohort	FS method	Best ML Model	# features	Acc	F1	Prec	Rec	Spec	AUC
UK	n/a	qMIDS	14	91.05	91.10	91.06	91.85	92.33	94.50
	No	LR	14	89.58	89.58	90.21	89.58	89.57	94.16
	No	XGB	15	91.49	91.50	91.84	91.49	91.53	94.34
	mRMR	LDA	14	91.13	91.15	91.69	91.13	91.77	95.76
IN-N	n/a	qMIDS	14	91.64	91.38	91.64	93.43	90.75	93.57
	No	XGB	15	90.09	89.66	92.10	90.09	89.31	95.76
	No	XGB	15	90.09	89.66	92.10	90.09	89.31	95.76
	mRMR	LR	2	93.51	93.36	94.74	93.51	93.29	94.82
IN-S	n/a	qMIDS	14	84.95	91.39	84.95	82.92	70.38	93.20
	No	LR	14	83.52	82.38	85.23	83.52	75.14	92.47
	No	XGB	15	92.29	92.25	94.53	92.29	92.45	92.73
	RFE	RF	10	95.33	94.94	96.72	95.33	94.20	97.10
CN	n/a	qMIDS	14	88.57	88.77	88.57	91.40	90.83	92.80
	mRMR	LR	5	92.57	92.88	95.00	92.57	96.83	99.20
	No	XGB	15	92.90	92.71	93.83	92.90	90.72	94.11
	RFE	LDA	14	93.14	93.40	95.33	93.14	96.86	98.00

As can be seen in Table 4, the best performing setup across all four datasets is observed by the early fusion of qMIDS with a ML classifier, for most of the performance metrics used. Without applying the early fusion architecture, the qMIDS method is achieving slightly better performance compared to the ML based setup, for most metrics. The improvement of the early fusion architecture compared to no fusion varies in terms of F1 from 0.52% in CN cohort up to 12.56% in IN-S cohort, and AUC is increased from 1.6% in UK cohort up to 4.63% in IN-S cohort. In terms of ML algorithms, LR and XGB were found to perform well in most datasets.

### Late fusion

The late fusion results for each of the four datasets are tabulated in Table 5. The qMIDS based and the best ML based results, i.e., without fusion, are highlighted in grey. The best results without feature selection (FS) and with the best FS are tabulated without background colour.

**Table 5:** Oral cancer identification results with Late fusion (results without fusion shown in grey).

Cohort	FS method	Best ML Model	# features	Acc	F1	Prec	Rec	Spec	AUC
UK	n/a	qMIDS	14	91.05	91.10	91.06	91.85	92.33	94.50
	No	LR	14	89.58	89.58	90.21	89.58	89.57	94.16
	No	LDA	14	90.46	90.47	90.83	90.46	90.46	94.97
	RFE	LDA	10	90.68	90.69	91.02	90.68	90.61	94.93
IN-N	n/a	qMIDS	14	91.64	91.38	91.64	93.43	90.75	93.57
	n/a	XGB	14	90.09	89.66	92.10	90.09	89.31	95.76
	No	XGB	14	90.13	89.89	91.75	90.13	89.67	96.00
	mRMR	XGB	3	90.89	90.75	92.38	90.89	90.71	96.40
IN-S	n/a	qMIDS	14	84.95	91.39	84.95	82.92	70.38	93.20
	No	LR	14	83.52	82.38	85.23	83.52	75.14	92.47
	No	SVM	14	93.24	93.10	95.26	93.24	92.90	99.27
	mRMR	LDA	3	93.33	93.12	95.49	93.33	93.47	99.17
CN	n/a	qMIDS	14	88.57	88.77	88.57	91.40	90.83	92.80
	mRMR	LR	5	92.57	92.88	95.00	92.57	96.83	99.20
	No	SVM	14	94.86	95.06	96.52	94.86	97.74	96.50
	mRMR	ET	3	93.71	93.90	95.71	93.71	96.29	97.20

As can be seen in Table 5, the best performing setup across all four datasets is observed by the late fusion of qMIDS with a ML classifier, for most of the performance metrics used. Without applying the late fusion architecture, the qMIDS method is achieving slightly better or comparable performance to the ML based results, for most metrics. The improvement of the late fusion architecture compared to no fusion varies in terms of F1 from 1.09% in IN-N cohort up to 10.74% in IN-S cohort, and AUC is increased from 0.64% in IN-N cohort up to 6.8% in IN-S cohort. In terms of ML algorithms, XGB, SVM and LR were found to perform well in most datasets.

Comparison between the early and the late fusion architectures shows that the late fusion is achieving higher performances across all/most metrics and across all/most datasets. Specifically, the difference between early and late fusion varies in terms of F1 from 0.58% in IN-S cohort up to 2.35% in CN cohort, and in terms of AUC from 0.24% in IN-N cohort up to 6.54% in IN-S cohort.

### Overfitting and Model Stability Assessment

Given the moderate-to-small size of some cohorts (Table 2), the stability of the best-performing early and late fusion models was further assessed using the standard deviation and 95% confidence interval (CI) of the AUC across the ten outer cross-validation folds, and using learning curves for each cohort and fusion architecture (Supporting Information, S1 Fig). Table 6 summarises the AUC variability for the best early and late fusion model in each of the four main cohorts; the corresponding table for all inter-dataset train/test combinations is provided in the Supporting Information (S1 Table). In all four cohorts, the standard deviation and 95% CI of the AUC across folds were narrow relative to the reported performance improvements, and learning curves showed converging (rather than diverging) training and validation performance as sample size increased, both consistent with the reported gains reflecting genuine signal rather than fold-specific overfitting; nonetheless, given the sample sizes involved, particularly for IN-S and CN, these results should be interpreted as supportive rather than definitive evidence against overfitting, and independent, prospectively-collected validation cohorts remain necessary (see Discussion, Limitations).

**Table 6:** AUC variability (standard deviation and 95% CI across ten-fold cross-validation) for the best early and late fusion model per cohort.

Cohort	Fusion	Best ML model	# features	FS method	AUC	Std AUC	95% CI AUC
UK	Early	LDA	14	mRMR	95.76	0.022	0.009
UK	Late	LDA	10	SVM-RFE	94.93	0.022	0.009
IN-N	Early	XGB	15	No	95.76	0.055	0.021
IN-N	Late	XGB	3	mRMR	95.36	0.059	0.023
IN-S	Early	RF	10	SVM-RFE	97.10	0.057	0.022
IN-S	Late	SVM	14	No	99.27	0.019	0.008
CN	Early	LDA	14	SVM-RFE	98.00	0.051	0.019
CN	Late	ET	3	mRMR	97.20	0.056	0.022

### Statistical significance testing

To assess whether the performance differences reported above exceed what could be expected from cross-validation fold variability alone, paired significance testing (fold-paired *t*-test on the per-fold AUC values) was performed between the best fusion model and the corresponding qMIDS-alone/no-fusion baseline in each cohort. Resulting *p*-values are summarised in Table 7; the corresponding table for all inter-dataset train/test combinations is provided in the Supporting Information (S2 Table). The comparisons reaching conventional significance (*p* < 0.05) were the early fusion model in the UK cohort, the early fusion model in the IN-S cohort, and the late fusion model in the CN cohort; the remaining comparisons, while showing consistent numerical improvements over the qMIDS-alone baseline, did not reach significance at the sample sizes available, particularly for the smaller IN-N, IN-S and CN cohorts. This is consistent with the overfitting assessment above and reinforces that the performance gains reported for the smaller cohorts should be treated as encouraging but preliminary until confirmed in larger, independent samples.

**Table 7:** Paired significance testing (fold-paired *t*-test on AUC) between the best fusion model and the qMIDS-alone/no-fusion baseline, per cohort.

Cohort	Fusion	Best ML model	# features	FS method	*p*-value
UK	Early	LDA	14	mRMR	0.005
UK	Late	LDA	10	SVM-RFE	0.637
IN-N	Early	XGB	15	No	0.140
IN-N	Late	XGB	3	mRMR	0.365
IN-S	Early	RF	10	SVM-RFE	0.018
IN-S	Late	SVM	14	No	0.109
CN	Early	LDA	14	SVM-RFE	0.691
CN	Late	ET	3	mRMR	0.008

We also note that the qMIDS-alone row already reported in Tables 4 and 5 (highlighted in grey) constitutes the requested simple baseline of thresholding the MI score alone, against which all fusion and no-fusion ML results are compared throughout the Results section.

Further results on inter-dataset analysis, presented below, showed that the proposed architectures have in most cases less than 2% drop in performance when the training and test data come from geographically and ethnically diverse patients.

### Direct comparison of early versus late fusion

The tests reported above compare each fusion architecture with the qMIDS-alone/no-fusion baseline, and therefore do not by themselves establish that late fusion outperforms early fusion. The two architectures were consequently also compared with each other directly. For every combination of classifier (the ten classifiers of Table 3), feature selection method (mRMR, SVM-RFE) and retained feature count (K∈{2,3,5,7,10,12,14}) – 140 matched configurations per cohort – the per-fold AUC values obtained with early and with late fusion were compared using a two-sided fold-paired *t*-test. Both archi*t*ectures share an identical classifier, feature selection method and feature count within each comparison, so that the difference observed is attributable to the fusion strategy itself. The results are summarised in Table 8; the full per-configuration results are provided in the Supporting Information (S3 Table).

**Table 8:** Direct comparison (two-sided fold-paired *t*-test on AUC) between the early and late fusion architectures, across all 140 matched classifier/feature-selection/feature-count configurations per cohort.

Cohort	Matched configs	Late sig. better	Early sig.	No sig. difference	Median ΔAUC (Late - Early), pp
UK	140	39	29	72	+0.15
IN-N	140	41	0	99	+1.61
IN-S	140	80	0	60	+4.06
CN	140	68	0	72	+3.67

The median AUC difference favoured late fusion in all four cohorts. In the IN-N, IN-S and CN cohorts late fusion was never significantly worse than early fusion in any of the 140 matched configurations, while significantly outperforming it in 41, 80 and 68 configurations respectively; in the UK cohort the picture is more balanced, with 39 configurations significantly favouring late fusion and 29 significantly favouring early fusion. When the comparison is instead restricted to the matched configuration corresponding to the best late fusion model of Table 6, the difference between the two architectures did not reach significance in the IN-N (*p* = 0.33), IN-S (*p* = 0.91) and CN (*p* = 0.83) cohorts, and significantly favoured early fusion in the UK cohort (*p* < 0.001). Late fusion is therefore better described as the more consistently reliable of the two architectures across classifier and feature-selection choices than as significantly superior to early fusion at its best-performing operating point, and the statements made elsewhere in this manuscript should be read in that qualified sense. The same matched comparison was also performed for the inter-dataset train/test combinations, using a two-sided bootstrap test on the external test cohort because each combination yields a single test set rather than ten cross-validation folds: across the 12 combinations and 120 matched configurations each, late fusion significantly outperformed early fusion in 397 of the 1,440 comparisons and early fusion significantly outperformed late fusion in none (S4 Table).

## Inter-dataset analysis

Further to the early vs late fusion architecture results, inter-dataset analysis was performed to investigate the robustness of the oral cancer diagnosis methods across geographically and ethnically diverse datasets, with one dataset used for training and another dataset for testing each time. The inter-dataset analysis results comparing qMIDS-based, no fusion, early fusion and late fusion for each train/test cohort are tabulated in Table 9.

**Table 9:** Inter-Dataset Oral Cancer Diagnosis Results (NF: No Fusion, EF: Early Fusion, LF: Late Fusion).

Cohort	FS method	Best ML Model	# features	Acc	F1	Prec	Rec	Spec	AUC
Train: UK Test: IN-N	n/a	qMIDS	14	91.64	91.38	91.64	93.43	90.75	93.57
	mRMR	kNN (NF)	10	77.08	77.09	77.17	77.08	77.18	79.65
	mRMR	AB (EF)	2	91.67	91.58	92.82	91.67	90.94	95.74
	mRMR	LR (LF)	3	91.67	91.58	92.82	91.67	90.94	95.30
Train: UK Test: IN-S	n/a	qMIDS	14	84.95	91.39	84.95	82.92	70.38	93.20
	SVM-RFE	kNN (NF)	10	84.85	84.66	84.64	84.85	78.79	87.19
	No	AB (EF)	3	96.97	96.93	97.10	96.97	93.94	98.35
	mRMR	GB (LF)	3	96.97	96.93	97.10	96.97	93.94	98.76
Train: UK Test: CN	n/a	qMIDS	14	88.57	88.77	88.57	91.40	90.83	92.80
	mRMR	SVM RBF (NF)	2	97.14	97.17	97.38	97.14	98.69	99.62
	No	SVM poly (EF)	3	94.29	94.29	94.29	94.29	92.46	97.73
	mRMR	RF (LF)	3	94.29	94.29	94.29	94.29	92.46	97.35
Train: IN-N Test: UK	n/a	qMIDS	14	91.05	91.10	91.06	91.85	92.33	94.50
	mRMR	ET (NF)	12	82.52	82.27	82.90	82.52	80.13	87.77
	mRMR	RF (EF)	14	89.32	89.36	89.94	89.32	90.39	92.23
	mRMR	LR (LF)	3	90.29	90.33	90.98	90.29	91.47	92.45
Train: IN-N Test: IN-S	n/a	qMIDS	14	84.95	91.39	84.95	82.92	70.38	93.20
	mRMR	kNN (NF)	12	84.85	84.14	85.17	84.85	74.24	81.40
	No	AB (EF)	15	96.97	96.93	97.10	96.97	93.94	99.17
	mRMR	XGB (LF)	3	96.97	97.00	97.22	96.97	98.48	99.59
Train: IN-N Test: CN	n/a	qMIDS	14	88.57	88.77	88.57	91.40	90.83	92.80
	No	AB (NF)	14	94.29	94.29	94.29	94.29	92.46	91.29
	SVM-RFE	ET (EF)	12	91.43	91.52	91.78	91.43	91.15	95.08
	SVM-RFE	kNN (LF)	3	91.43	91.66	93.27	91.43	96.07	97.35
Train: IN-S Test: UK	n/a	qMIDS	14	91.05	91.10	91.06	91.85	92.33	94.50
	No	SVM RBF (NF)	14	83.50	83.27	83.86	83.50	81.22	90.90
	mRMR	AB (EF)	7	90.29	90.33	90.73	90.29	91.14	91.72
	mRMR	GB (LF)	3	87.70	87.75	88.69	87.70	89.13	92.61
Train: IN-S Test: IN-N	n/a	qMIDS	14	91.64	91.38	91.64	93.43	90.75	93.57
	SVM-RFE	SVM RBF (NF)	2	68.75	66.66	73.21	68.75	66.73	68.09
	mRMR	AB (EF)	10	91.67	91.58	92.82	91.67	90.94	97.39
	SVM-RFE	kNN (LF)	3	93.75	93.71	94.42	93.75	93.21	94.78
Train: IN-S Test: CN	n/a	qMIDS	14	88.57	88.77	88.57	91.40	90.83	92.80
	No	SVM RBF (NF)	14	88.57	88.23	88.60	88.57	79.99	96.59
	mRMR	ET (EF)	14	94.29	94.29	94.29	94.29	92.46	95.45
	mRMR	kNN (LF)	3	91.43	91.66	93.27	91.43	96.07	95.45
Train: CN Test: UK	n/a	qMIDS	14	91.05	91.10	91.06	91.85	92.33	94.50
	SVM-RFE	LDA (NF)	10	81.23	81.30	82.06	81.23	82.28	88.92
	mRMR	LR (EF)	2	87.38	87.43	88.14	87.38	88.55	94.06
	mRMR	AB (LF)	3	89.97	89.89	90.15	89.97	88.56	94.67
Train: CN Test: IN-N	n/a	qMIDS	14	91.64	91.38	91.64	93.43	90.75	93.57
	mRMR	LR (NF)	5	70.83	67.64	81.30	70.83	68.30	79.48
	SVM-RFE	LR (EF)	3	70.83	70.21	71.92	70.83	69.69	77.74
	SVM-RFE	LDA (LF)	3	91.67	91.58	92.82	91.67	90.94	93.48
Train: CN Test: IN-S	n/a	qMIDS	14	84.95	91.39	84.95	82.92	70.38	93.20
	mRMR	LR (NF)	3	81.82	80.56	82.42	81.82	68.18	81.82
	No	LR (EF)	15	84.85	84.14	85.17	84.85	74.24	88.43
	mRMR	GB (LF)	3	96.97	96.93	97.10	96.97	93.94	98.35

As can be seen in Table 9, the use of different datasets for training and testing does not significantly affect the performance of oral cancer diagnosis. In specific, for the UK cohort the best observed performance at the inter-dataset setup is 1.17% of F1 score lower than when using only the UK cohort for training/testing and 1.09% of AUC score lower. For the IN-N cohort the best observed performance at the inter-dataset setup is 1.06% of F1 score higher and 0.99% of AUC score higher, while for the IN-S cohort the best observed performance at the inter-dataset setup is 2.06% of F1 score higher and 0.32% of AUC score higher. For the CN cohort the best observed performance at the inter-dataset setup is 0.77% of F1 score lower and 1.47% of AUC score lower.

For the IN-N cohort the best observed performance when IN-S is used for training is 3.64% of F1 score higher than when using IN-N only and 3.19% of AUC score higher. For the IN-S cohort the best observed performance when IN-N is used for training is 1.23% of F1 score lower than when using IN-S only and 1.88% of AUC score lower.

## Discussion

### Beyond the performance gain: what the fusion results indicate biologically

The qMIDS malignancy index is a fixed, closed-form combination of the log_2_ fold-change of 14 FOXM1-network genes against a healthy reference distribution, in which every target gene contributes additively and a single population-derived threshold (MI = 4) is used to call a sample malignant. The finding that a learned meta-classifier operating on the same 14 transcripts recovers additional discriminative information (Tables 4–5) indicates that the FOXM1-driven oncogenic signal in these tissues is not purely additive: the diagnostic information carried jointly by pairs and higher-order combinations of the 14 genes exceeds what a single linear index can represent. This is consistent with the underlying biology of the panel, in which FOXM1 sits at the centre of an interconnected transcriptional program governing proliferation, genomic instability and stromal/immune modulation [12], so that the target genes are expected to be co-regulated rather than independent. The largest gains from fusion were observed in the cohort with the most heterogeneous case-mix and smallest sample size (IN-S), where a fixed threshold calibrated on other populations is most likely to be miscalibrated; the ML layer effectively re-weights and re-thresholds the FOXM1 signature to the local distribution of the transcripts without altering the underlying gene panel or the biology it measures. The principal insight of the fusion analysis is therefore less that late fusion boosts performance in isolation, and more that a single, fixed molecular threshold is an incomplete description of a multi-gene oncogenic program, and that this gap can be partially closed statistically, using the existing 14-gene assay, rather than by discovering new genes.

### Clinical and translational significance

The current diagnostic pathway for oral potentially malignant disorders (OPMD) and OSCC relies heavily on histopathological assessment, which is subject to inter-observer variability and cannot reliably predict which OPMD lesions will progress to malignancy [1]. qMIDS was designed to address this gap, offering a quantitative molecular readout (see Materials and methods) that does not depend on subjective histological interpretation and can support earlier risk stratification and intervention. A key practical constraint of any fixed diagnostic formula, however, is that it requires re-derivation of its reference distribution and threshold whenever it is deployed on a new population, sample-handling protocol or instrument, which is costly and slow. Our results show that this recalibration can instead be achieved with a lightweight, retrainable ML layer applied on top of the existing 14-gene assay, without redesigning the underlying gene panel, biochemistry, or the interpretable qMIDS score itself, which remains available to the clinician alongside the fused prediction. The inter-dataset experiments (Table 9) show that some degree of this adaptation is achievable even when the training and deployment populations differ substantially in ethnicity and geography, with performance drops generally under 2% in most, though not all, train/test combinations. Practically, this supports a translational pathway in which a single qMIDS assay could be deployed across new clinical sites, with a small, locally-collected calibration set used to retrain the fusion layer rather than a full re-validation study of the underlying molecular test; by improving sensitivity relative to the fixed-threshold baseline in the IN-S (82.92% to 93.33%) and CN (91.40% to 94.86%) cohorts, the fused models may also reduce false-negative misclassification and support more reliable early intervention in routine care. This is particularly relevant for oral cancer, where the burden and the settings in which OPMD/OSCC screening is most needed (e.g., India, China, and other high-incidence regions) can differ considerably from the population in which a test was originally developed and validated.

### Interpreting the inter-dataset transferability patterns

The variability observed across the different train/test combinations in Table 9 is itself informative. Several non-mutually-exclusive factors are likely to contribute to why certain train-test combinations (e.g., those involving the Indian cohorts) transfer more successfully than others: (i) *biological and demographic similarity* – population pairs that share more comparable environmental exposures, lifestyle risk factors (e.g., tobacco/betel-quid use) or tumour biology are expected to show more aligned FOXM1-network dysregulation patterns, and therefore better model transfer; (ii) *tissue-processing compatibility* – differences in sample preservation and handling between sites can shift the expression distributions captured by RT-qPCR, and train/test pairs processed under more similar protocols would be expected to transfer more consistently; and (iii) *technical heterogeneity across cohorts* – differences in RNA extraction chemistry, reagent lots, or amplification efficiency between laboratories can imprint cohort-specific technical signatures that are difficult to fully disentangle from biological signal in retrospective, non-batch-corrected data (see RT-qPCR Data Preprocessing).

A related observation is that early fusion occasionally outperforms late fusion in the inter-dataset setting in terms of raw AUC, although in a direct matched comparison across the same train/test combinations early fusion did not significantly outperform late fusion in any of the 1,440 configurations tested (S4 Table), in contrast to the same-cohort setting where late fusion was consistently the best architecture. We interpret this as follows: early fusion allows the feature selection and classification steps to learn direct interactions between the MI score and the individual gene features, which can produce a more robust joint representation when the train and test distributions differ; late fusion, by contrast, trains its base classifier and meta-classifier on two representations that are optimised independently, which can make it more sensitive to error propagation when the base classifier’s own calibration shifts under distribution change. Late fusion would be expected to have the advantage when the two modalities being fused (gene features and MI) have very different scales, when sample sizes are small (favouring separately pre-trained, lower-variance base models), or when one modality is more reliable than the other in a given cohort. Together, these results indicate that neither fusion architecture dominates the other under population shift, and that the appropriate choice may depend on the degree and nature of the distribution shift between the training and deployment populations.

Practically, these transferability results suggest that qMIDS-based ML models should not be assumed to generalise uniformly to a new population without prior evaluation. We recommend that any deployment in a new population, laboratory, or sample-handling protocol be preceded by a population-specific validation step using a small, locally representative sample set, both to confirm performance and, if necessary, to retrain the late-fusion layer as described above; because false-negative misclassification could delay cancer diagnosis, this local validation step is a clinically important safeguard rather than an optional refinement. Where such validation confirms good transfer (as observed for several train/test pairs here), cross-population deployment appears feasible without redeveloping the underlying assay. More broadly, these findings support the value of region-aware validation pipelines and of incorporating multi-population training data when the eventual goal is a single, globally deployable classifier.

### Generalisability beyond oral cancer

The FOXM1 oncogenic program probed by qMIDS is not specific to the oral cavity: FOXM1 is among the most consistently upregulated transcription factors across at least 39 cancer types and is associated with poor prognosis in most of them [12]. This raises the possibility that the same class of FOXM1-responsive genes could be re-purposed as an adjunct to diagnostics for other cancers with a shared FOXM1-driven proliferative/genomic-instability phenotype, particularly other epithelial cancers with comparable molecular dysregulation patterns, subject to independent validation in those tissue types. More generally, the computational architecture evaluated here – pairing a mechanistically-anchored, clinically-interpretable molecular index with an adaptive ML meta-classification layer – is not specific to qMIDS or to FOXM1: any diagnostic test that reduces a multi-gene or multi-marker assay to a single validated index (for example, other RT-qPCR- or microarray-based recurrence/risk scores used in other cancers) could in principle be paired with a similar late-fusion layer to gain population-level adaptability while retaining the interpretability and regulatory track record of the original index. We therefore view the present study’s main contribution as evidence for, and a template of, a general “interpretable index + adaptive ML layer” strategy for improving the portability of molecular cancer diagnostics across diverse populations, in addition to its specific application to oral cancer.

### Limitations

Several limitations should be considered when interpreting these results. First, the cohorts used here, while geographically and ethnically diverse, remain moderate in size (33–309 samples per cohort), and the smaller Indian (IN-S, n = 33) and Chinese (CN, n = 35) cohorts in particular limit the precision with which performance differences can be estimated; some of the AUC improvements reported to three significant figures for these cohorts are within the confidence intervals reported in the Overfitting and Model Stability Assessment subsection and did not reach statistical significance (Table 7), and should therefore be interpreted as preliminary rather than conclusive. Second, although nested cross-validation, standard deviation/CI reporting and learning-curve analysis did not reveal evidence of substantial overfitting, these are not a substitute for prospective, independently-collected validation cohorts. Third, the cohorts were analysed separately by geography; pooling all available samples prior to train/test partitioning may further improve the generalisability of a single classifier across ethnicities, at the cost of the population-specific transferability analysis presented here, and represents a useful direction for future work as sample sizes grow. Finally, extension of the fusion architectures to other FOXM1-associated cancers, and prospective clinical validation of the late-fusion qMIDS model, are needed before the translational and cross-cancer claims above can be considered confirmed rather than hypothesis-generating.

## Conclusion

Early and late fusion architectures of the qMIDS method with ML classifiers for oral cancer diagnosis from gene expression data were presented. The evaluation was performed on four datasets from UK, India and China and the evaluation results showed that late fusion outperformed the other evaluated setupson aggregate for most of the metrics evaluated. The best-performing late fusion models were LDA (UK), XGB (IN-N), SVM (IN-S) and Extra Trees (CN). Relative to the qMIDS-alone baseline, these improved AUC by 0.47, 2.83, 6.07 and 4.40 percentage points in the UK, IN-N, IN-S and CN cohorts respectively, and improved F1 by 1.73 (IN-S) and 6.29 (CN) percentage points while remaining within 0.7 percentage points of qMIDS alone in the UK and IN-N cohorts. Relative to the corresponding ML-without-fusion baseline, late fusion improved F1 by up to 10.74 and AUC by up to 6.8 percentage points (both in the IN-S cohort). Further analysis using different datasets for training and test showed robustness of the proposed architectures, demonstrating the transferability of the approach, although early fusion occasionally outperformed late fusion under population shift in terms of raw AUC, though never significantly so (S4 Table), likely reflecting early fusion’s ability to learn direct interactions between the MI score and gene features when train and test distributions differ (see Discussion). Beyond the performance improvement itself, these results indicate that the FOXM1-driven oncogenic program captured by the 14-gene qMIDS panel carries diagnostic information that a single linear index cannot fully exploit, and that this residual information can be recovered with a lightweight, locally-retrainable ML layer rather than by redesigning the underlying assay. This “interpretable index + adaptive ML layer” strategy may offer a practical route to deploying qMIDS, and potentially other FOXM1-driven or single-index molecular diagnostics, across new and diverse patient populations with reduced re-validation burden. Given the moderate size of some of the cohorts studied, further validation on larger, prospectively-collected and/or pooled multi-ethnic datasets is required before these architectures can be considered ready for clinical deployment.

## Supporting information

S1 Fig Learning curves for the best-performing early and late fusion model, per cohort and per inter-dataset train/test combination. 32 panels; referenced in the Results (subsection “Overfitting and Model Stability Assessment”).
(TIF)

S2 Fig Calibration curves for the best-performing base and meta-classifier models, per cohort and per inter-dataset train/test combination. 32 panels; referenced in the Materials and methods (Late Fusion subsection).
(TIF)

S1 Table AUC variability (standard deviation and 95% CI across ten-fold cross-validation) for the best early and late fusion model, for all inter-dataset train/test cohort combinations. Referenced in the Results (subsection “Overfitting and Model Stability Assessment”).
(XLSX)

S2 Table Paired significance testing (*t*-test on AUC) between the best fusion model and the qMIDS-alone/no-fusion baseline, for all inter-dataset train/test cohort combinations. Referenced in the Results (subsection “Statistical Significance Testing”).
(XLSX)

S3 Table Direct comparison (two-sided fold-paired *t*-test on AUC) between the early and late fusion architectures, for every matched combination of classifier, feature selection method and retained feature count. 140 matched configurations per cohort; referenced in the Results (subsection “Direct Comparison of Early versus Late Fusion”).
(XLSX)

S4 Table Direct comparison (two-sided bootstrap test on AUC) between the early and late fusion architectures in the inter-dataset setting, summarised per train/test cohort combination. 120 matched configurations per combination; referenced in the Results (subsection “Direct Comparison of Early versus Late Fusion”) and in the Discussion.
(XLSX)

S1 Data Oral Cancer Datasets. Consists of four datasets (UK, IN-N, IN-S, CN) quantitative information of 14 target genes (HOXA7, CENPA, NEK2, DNMT1, INHBA, FOXM1, TOP2A, BIRC5, MMP13, CXCL8, NR3C1, IVL, CBX7, and S100A16) measured by RT-qPCR from each sample.
(XLSX)
